# Single-molecule real-time sequencing identifies massive full-length cDNAs and alternative-splicing events that facilitate comparative and functional genomics study in the hexaploid crop sweet potato

**DOI:** 10.7717/peerj.7933

**Published:** 2019-11-15

**Authors:** Na Ding, Huihui Cui, Ying Miao, Jun Tang, Qinghe Cao, Yonghai Luo

**Affiliations:** 1 Fujian Provincial Key Laboratory of Plant Functional Biology, College of Life Sciences, Fujian Agriculture and Forestry University, Fuzhou, Fujian, China; 2 School of Life Sciences, Jiangsu Normal University, Xuzhou, Jiangsu, China; 3 Jiangsu Xuhuai Regional Xuzhou Institute of Agricultural Sciences, Xuzhou, Jiangsu, China; 4 Key Laboratory of Biology and Genetic Improvement of Sweetpotato, Ministry of Agriculture, Xuzhou, Jiangsu, China

**Keywords:** Single-molecular real-time sequencing, Comparative transcriptome analysis, Alternative splicing, Sweet potato

## Abstract

**Background:**

Sweet potato (*Ipomoea batatas* (L.) Lam.) is one of the most important crops in many developing countries and provides a candidate source of bioenergy. However, neither a complete reference genome nor large-scale full-length cDNA sequences for this outcrossing hexaploid crop are available, which in turn impedes progress in research studies in *I. batatas* functional genomics and molecular breeding.

**Methods:**

In this study, we sequenced full-length transcriptomes in *I. batatas* and its diploid ancestor *I. trifida* by single-molecule real-time sequencing and Illumina second-generation sequencing technologies. With the generated datasets, we conducted comprehensive intraspecific and interspecific sequence analyses and experimental characterization.

**Results:**

A total of 53,861/51,184 high-quality long-read transcripts were obtained, which covered about 10,439/10,452 loci in the *I. batatas*/*I. trifida* genome. These datasets enabled us to predict open reading frames successfully in 96.83%/96.82% of transcripts and identify 34,963/33,637 full-length cDNA sequences, 1,401/1,457 transcription factors, 25,315/27,090 simple sequence repeats, 1,656/1,389 long non-coding RNAs, and 5,251/8,901 alternative splicing events. Approximately, 32.34%/38.54% of transcripts and 46.22%/51.18% multi-exon transcripts underwent alternative splicing in *I. batatas*/*I. trifida*. Moreover, we validated one alternative splicing event in each of 10 genes and identified tuberous-root-specific expressed isoforms from a starch-branching enzyme, an alpha-glucan phosphorylase, a neutral invertase, and several ABC transporters. Overall, the collection and analysis of large-scale long-read transcripts generated in this study will serve as a valuable resource for the *I. batatas* research community, which may accelerate the progress in its structural, functional, and comparative genomics studies.

## Introduction

Sweet potato (*Ipomoea batatas* (L.) Lam.) is the seventh most important crop in the world and it ensures food supply and safety in many developing countries. *I. batatas* is a hexaploid plant with a complex and heterozygous genome (2n = 6 × = 90, 3–4 gigabase pairs in genome size (Magoon, Krishnan & Vijaya, 1970; Ozias-Akins & Jarret, 1994)). A preliminary genome estimate has revealed two genome polyploidization events occurring about 0.8 and 0.5 million years ago (Yang et al., 2017). Nevertheless, the complete reference genome of *I. batatas* remains lacking, which hinders the progress in molecular dissections of its evolutionary scenario and agronomically important traits. Moreover, *I. batatas* is a self-incompatible and thus obligate, outcrossing species (Martin, 1965). It is almost impossible to develop typical mapping populations such as F2 and recombinant inbred lines for constructing high-density linkage maps and classical genetic analyses. To date, no successful investigation in forward genetics (i.e., quantitative trait locus mapping and subsequently map-based cloning) of *I. batatas* has been reported. Therefore, RNA sequencing (i.e., RNA-seq, whole transcriptome shotgun sequencing (Wang, Gerstein & Snyder, 2009)) has been widely used as an attractive alternative to whole genome sequencing for gene mining in *I. batatas* (Schafleitner et al., 2010; Wang et al., 2010; Nurit et al., 2013). However, all reported transcriptomes in *I. batatas* were derived from second-generation sequencing platforms, which generate relatively short reads (i.e., hundreds of base pairs per read) and are disadvantageous in obtaining full-length transcripts (Koren et al., 2012). To date, the collection and analysis of large-scale full-length cDNA sequences have not been done in *I. batatas*, which is fundamental to its structural and functional genomics studies.

*Ipomoea trifida* (H.B.K.) G. Don has been considered as the diploid ancestor of *I. batatas* and accumulative evidence supports this hypothesis (Srisuwan, Sihachakr & Siljak-Yakovlev, 2006; Wu et al., 2018). Nevertheless, the evolutionary scenario underlying the origin and domestication of *I. batatas* remains unclear. Unlike *I. batatas*, *I. trifida* does not form tuberous roots, and thus comparative analysis of *I. batatas* and *I. trifida* may provide insights into the evolution and domestication of *I. batatas*. Although the reference genome of *I. trifida* becomes available recently (Wu et al., 2018) and short-read transcriptomes of *I. trifida* have been analyzed in a few projects (Cao et al., 2016; Ponniah et al., 2017), no study involving the large-scale collection and analysis of full-length cDNA sequences in *I. trifida* has been reported.

Long-read or full-length cDNA sequences are fundamental to structural and functional genomics studies. First, they provide complete information of transcribed sequences, which are required to gene function analyses. Second, they facilitate accurate predictions of gene models (i.e., to define proper orientation, order, and boundary of exons). Third, they may be utilized in validating or correcting the scaffold assembly in genome sequencing projects. Fourth, they are particularly useful to analyze alternative splicing of transcript isoforms, which is important to increase transcriptome diversity and adaptation potential of an organism. In the past, collecting full-length cDNA sequences was expensive, labor intensive, and time consuming (Seki et al., 2002; Shoshi et al., 2003). The advent of a third-generation sequencing platform (i.e., single-molecule real-time (SMRT) sequencing) has revolutionized DNA sequencing and thus genome/transcriptome studies  (Eid et al., 2010). Long reads of up to 20-kb in size, albeit with a relatively high error rate, can be produced by SMRT sequencing (Roberts, Carneiro & Schatz, 2013; Au et al., 2013). Today, high-throughput sequencing combining second-generation sequencing (to generate short reads with high base quality) and SMRT sequencing (to produce long reads with a relatively high error rate) has become an attractive option in genome and transcriptome studies (Au et al., 2013; Sharon et al., 2013; Xu et al., 2015). In the present study, we performed SMRT sequencing to generate large-scale full-length or long-read transcripts from *I. batatas* and *I. trifida*, respectively. Comprehensive intraspecific and interspecific sequence analyses were conducted, which has provided a valuable resource for the research community to exploit the origin of *I. batatas*.

## Materials & Methods

### Plant material and RNA preparation

**Figure 1:**
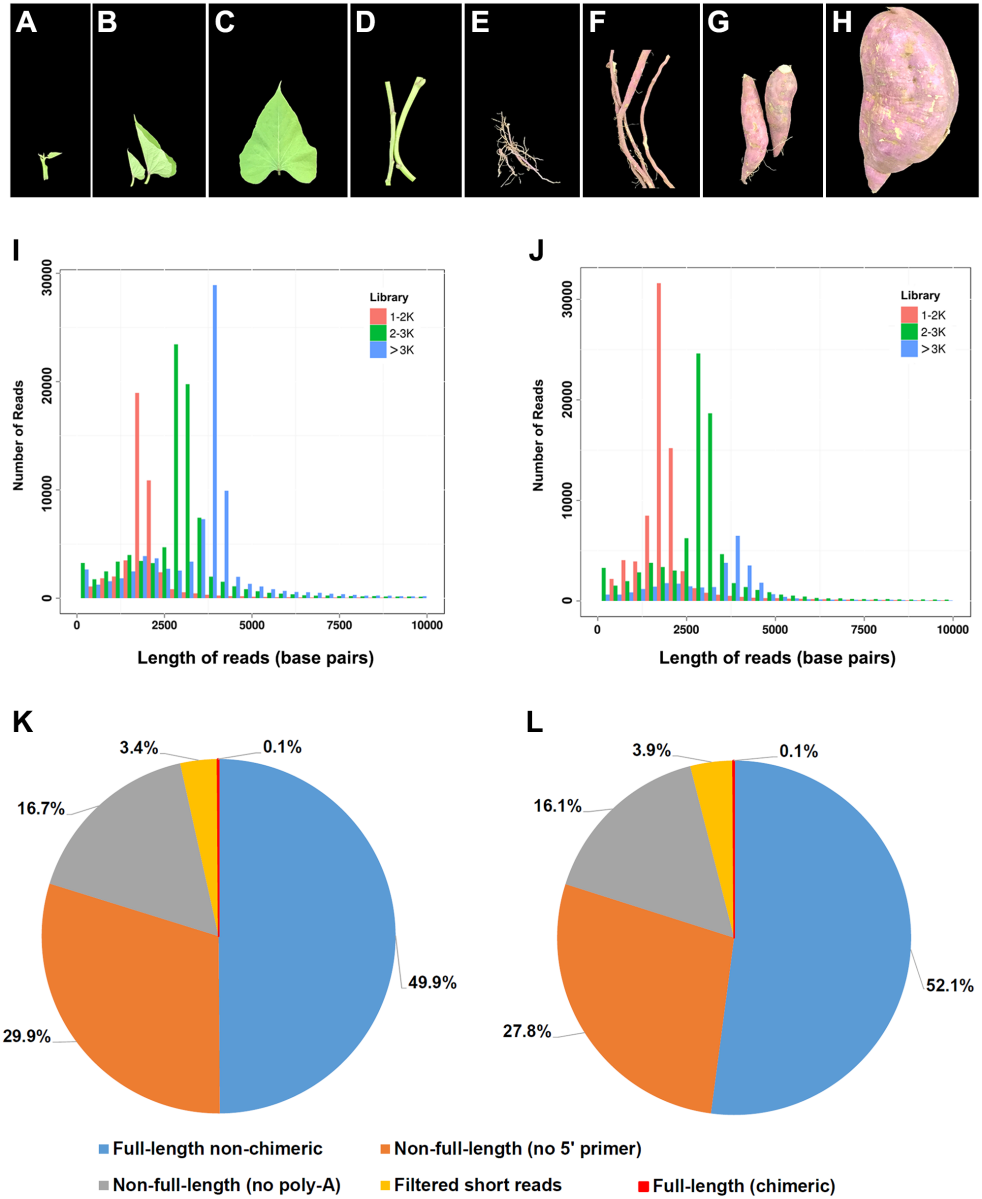
Plant materials used in this study and summary of PacBio RS II single-molecule real-time (SMRT) sequencing. (A–H) Photos showing the developmental stages and overall morphology of eight tissues in *I. batatas* used for SMRT sequencing in this study. (A) Young leaves; (B) mature leaves; (C) apical shoots; (D) mature stems; (E) fibrous roots; (F) initiating tuberous roots; (G) expanding tuberous roots; (H) mature tuberous roots. The photos were adopted from our previous report (Ding et al., 2017). Number and length distributions of 220,035 reads in *I. batatas* (I) and 195,188 reads in *I. trifida* (J) from different PacBio libraries (fractionated size: 1–2, 2–3, >3 kb); Proportion of different types of PacBio reads in *I. batatas* (K) and *I. trifida* (L).

*Xushu18*, one of the most widely cultivated *I. batatas* varieties in China, was selected for transcriptome sequencing in this study. Eight tissues of young leaves, mature leaves, apical shoots, mature stems, fibrous roots, initiating tuberous roots, expanding tuberous roots, and mature tuberous roots from one individual were collected and pooled together in approximately equivalent weights (Figs. 1A–1H). Similarly, tissues of young leaves, mature leaves, shoots, stems, and roots of a diploid *I. trifida* plant were collected and pooled. Collected samples were frozen in liquid nitrogen immediately after collection and stored at −80 °C until use.

Total RNAs were extracted using Tiangen RNA preparation kits (Tiangen Biotech, Beijing, China) following the provided protocol. RNA quality and quantity were determined using a Nanodrop ND-1000 spectrophotometer (NanoDrop Technologies, Wilmington, DE, USA) and a 2100 Bioanalyzer (Agilent Technologies, Palo Alto, CA, USA). Qualified RNA samples were subsequently used in constructing PacBio cDNA or RNA-seq libraries.

### PacBio cDNA library construction and SMRT sequencing

cDNA was synthesized using a SMARTer PCR cDNA Synthesis Kit, optimized for preparing full-length cDNA (Takara Clontech Biotech, Dalian, China). Size fractionation and selection (1–2 kb, 2–3 kb, and >3 kb) were performed using the BluePippin™ Size Selection System (Sage Science, Beverly, MA, USA). The SMRT bell libraries were constructed with the Pacific Biosciences DNA Template Prep Kit 2.0. SMRT sequencing was then performed on the Pacific Bioscience RS II platform using the provided protocol.

### Illumina RNA-Seq library construction and sequencing

The RNA-Seq libraries were constructed using a NEBNext® Ultra™ RNA Library Prep Kit for Illumina® (NEB, Beverly, MA, USA), following the manufacturer’s protocol. Qualified libraries were applied to transcriptome sequencing using an Illumina Hiseq 2500 (Illumina, San Diego, CA, USA) to generate 150-bp paired-end sequence reads (2 × 150 bp). High-throughput sequencing reported in this study was performed in the Biomarker Technology Co. (Beijing, China).

### Quality filtering and error correction of SMRT long reads

The SMRT subreads were filtered using the standard protocols in the SMRT Analysis software suite (http://www.pacificbiosciences.com), and reads of insert (ROIs) were obtained using the standard protocols in the SMRT Analysis software suite (parameters: minFullPass=0, minPredictedAccuracy=75). After examining for poly(A)signals and 5′ and 3′ adaptors, full-length and non-full-length cDNA reads were recognized. Consensus isoforms were identified using the algorithm of iterative clustering for error correction and further polished to obtain high-quality consensus isoforms. The raw Illumina reads were filtered to remove adaptor sequences, ambiguous reads with ’N’ bases, and low-quality reads. Afterward, error correction of low-quality isoforms was conducted using the Illumina reads with the software proovread 2.13.841 (parameters: –coverage=50 –overwrite, –no-sampling) (Hackl et al., 2014). Redundant isoforms were then removed to generate a high-quality transcript dataset for each species (i.e., Ib53861 for *I. batatas* and It51184 for *I. trifida*, respectively) using the program CD-HIT 4.6.142 (parameters: -c 0.99 -T 6 -G 0 -aL 0.90 -AL 100 -aS 0.99 -AS 30 -o) (Li & Godzik, 2006).

### Functional assignment of transcripts

Functional annotations were conducted by using BLASTX (cutoff *E*-value ≤ 1e−5) against different protein and nucleotide databases of COG (clusters of orthologous Groups; https://www.ncbi.nlm.nih.gov/COG/), GO (gene ontology; http://geneontology.org/), KEGG (kyoto encyclopedia of genes and genomes; https://www.kegg.jp/), Pfam (a database of conserved protein families or domains; http://pfam.xfam.org/), Swiss-prot (a manually annotated, non-redundant protein database; https://www.uniprot.org/), TrEMBL (an automatically annotated protein database; https://www.uniprot.org/), and NR (NCBI non-redundant proteins; https://www.ncbi.nlm.nih.gov/). For each transcript in each database searching, the functional information of the best matched sequence was assigned to the query transcript.

### Predictions of open reading frames and simple sequence repeats

To predict putative open reading frames (ORFs) in transcripts, we used the package TransDecoder v2.0.1 (https://transdecoder.github.io/) to define coding sequences (CDS). The predicted CDS were searched and confirmed by BLASTX (*E*-value ≤1e−5) against the protein databases of NR, SWISS-PROT, and KEGG. Those transcripts containing complete ORFs as well as 5′- and 3′-UTR (untranslated regions) were designated as full-length transcripts. To identify putative simple sequence repeats (SSRs) in our sequences, the tool MISA (MIcroSAtellite identification tool; http://pgrc.ipk-gatersleben.de/misa) was employed. Only transcripts that were ≥500 bp in size were included in SSR detection.

### Identification of transcription factor gene families

This was done according to our previous publication (Ding et al., 2017). Briefly, for each transcription factor gene family, the Hidden Markov Model (HMM) profile of the Pfam domain (when available) was downloaded from the Pfam database (http://pfam.xfam.org) and used as a query to survey all predicted proteins out of our transcript datasets using HMMER (http://www.hmmer.org). When no HMM profile was available for a gene family, all protein sequences belonging to the gene family in *A. thaliana* were downloaded (http://www.arabidopsis.org) and used as query sequences to search for our predicted protein datasets using BLASTP (*E*-value ≤1e−10). One redundant sequence was removed if two proteins shared the identity of amino acids equal to or larger than 97%. All identified non-redundant proteins were confirmed the existence of featured domains by searching the NCBI Conserved Domain Database (https://www.ncbi.nlm.nih.gov/Structure/cdd/wrpsb.cgi). The confirmed protein sequences as well as their corresponding transcripts were compiled (Only those gene families containing more than 10 members in at least one of our transcriptomes were presented).

### Prediction of long non-coding RNAs

To sort non-coding RNAs from putative protein-coding ones, we employed each of four computational approaches including CPC (Kong et al., 2007), CNCI (Liang et al., 2013), Pfam (Finn et al., 2016), and CPAT (Wang et al., 2013). Putative protein-coding RNAs were filtered out using a minimum length and exon number threshold according the instructions of programs. For each species, the intersection of the four resulting lists were obtained as final lncRNA candidates.

### Identification and validation of alternative splicing

To identify alternative splicing (AS) events, all transcripts of Ib53861 and It51184 were mapped to the the genomic contigs in *I. batatas* (Yang et al., 2017) and *I. trifida* (Wu et al., 2018), respectively, by using the program GMAP (Wu & Watanabe, 2005). The tool AStalavista v3.2 was employed to identify putative AS events (Foissac & Sammeth, 2007). Subsequently, 16 of AS events were selected and 10 of them were successfully confirmed by RT-PCR. Total RNA was isolated from the eight tissues in a *I. batatas* cultivar (*Xushu22*) as described above. The cDNA was synthesized using a cDNA Synthesis Kit (ProbeGene, China) and used as the template for PCR amplification. Afterward, PCR products were visualized in agarose gel.

## Results

### SMRT sequencing and generation of full-length transcriptomes

**Figure 2:**
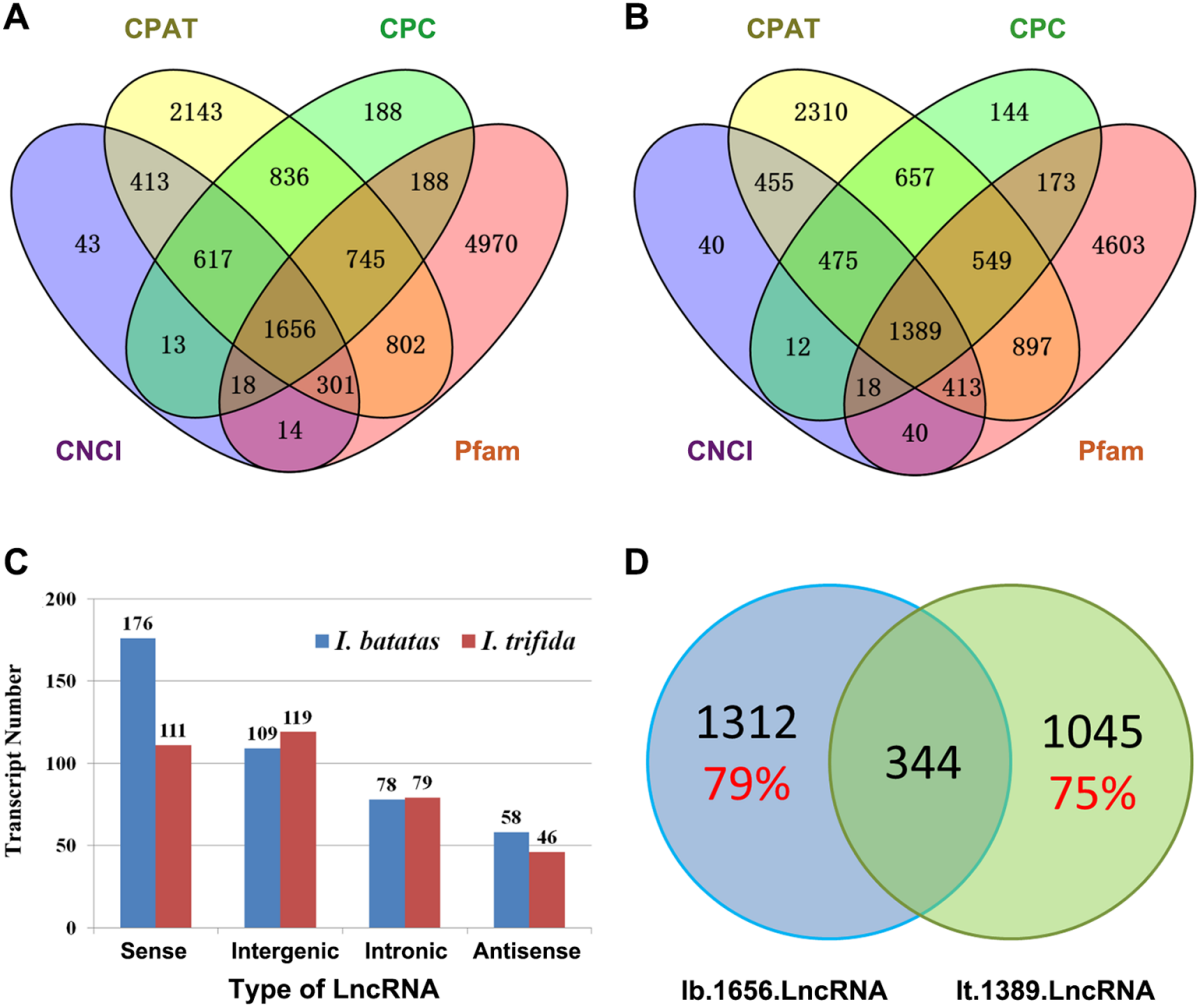
Analysis of putative long noncoding RNA (lncRNA). Venn Diagrams of lncRNAs predicted from Ib53861 (A) and It51184 (B) by four programs (CPAT, CPC, CNCI, and Pfam). (C) Types and numbers of lncRNAs that could be clarified in our analysis. (D) Homolog relationship of predicted lncRNAs between *I. batatas* and *I. trifida*.

To obtain large-scale long-read transcripts for *I. batatas* and *I. trifida*, respectively, SMRT sequencing was performed using a Pacific RSII sequencing platform. Eight different tissues collected from a single plant of each species were pooled and used in mRNA extraction. Three size-fractionated, full-length cDNA libraries were constructed and subsequently sequenced in four SMRT cells (Figs. 1I and 1J; 1–2 kb for one cell, 2–3 kb for two cells, and >3 kb for one cell). In *I. batatas*, we obtained 220,035 reads of the insert (total bases: 701,923,565), which included 49.9% of full-length non-chimeric and 46.6% of non-full-length reads (Table 1, Fig. 1K), whereas in *I. trifida*, 195,188 reads of the insert (total bases: 527,497,043) were generated, of which 52.1% and 43.9% were full-length non-chimeric and non-full-length reads, respectively (Table 1, Fig. 1L).

**Table 1:** Summary of PacBio sequencing in this study.

	*I. batatas*	*I. trifida*
Reads of insert of PacBio sequencing	220,035	195,188
Bases of insert of PacBio sequencing (bp)	701,923,565	527,497,043
Reads of Illumina sequencing for correction	71,360,785	39,372,131
Bases of Illumina sequencing for correction (bp)	17,972,706,252	11,772,267,169
Number of non-full-length PacBio reads	102,510	85,680
Number of full-length non-chimeric PacBio reads	109,814	101,630
Average length of full-length non-chimeric PacBio reads (bp)	8,641	8,488
Number of non-redundant transcripts after correction	53,861	51,184
N50 of non-redundant transcripts after correction (bp)	2,933	2,642
Mean of non-redundant transcripts after correction (bp)	2,421	2,190
Number of non-redundant full-length transcripts after correction	34,963	33,637

Given that SMRT sequencing generates a high error rate, it is necessary to perform error correction, which includes self-correction by iterative clustering of circular-consensus reads and correction with high-quality Illumina short reads. To this end, cDNA libraries were prepared from the same samples that were used for SMRT sequencing, and deep RNA sequencing was conducted using an Illumina Hiseq2500 platform. A total of 71,360,785 and 39,372,131 clean reads (total bases: 17,972,706,252 and 11,772,267,169, respectively) were obtained and used to correct the SMRT reads in *I. batatas* and *I. trifida*, respectively (Table 1). After error correction, redundant transcripts were removed. Finally, we obtained 53,861 transcripts for *I. batatas* (named as Ib53861; N50: 2,933 bp; mean: 2,421 bp) and 51,184 for *I. trifida* (named as It51184; N50: 2,642 bp; mean: 2,190 bp). Those transcripts containing complete coding sequences (CDSs) as well as 5′- and 3′-UTR (untranslated regions) were defined as full-length transcripts. Approximately 34,963 and 33,637 full-length transcripts were identified for *I. batatas* (named as Ib34963) and *I. trifida* (named as It33637), respectively (File S1).

### Basic sequence analysis of the full-length transcriptomes

The transcripts of Ib53861 and It51184 were functionally assigned and classified according to sequence similarities using BLASTx or tBLASTx (*E*-value ≤1e−5) against different protein and nucleotide databases. Overall, we successfully identified homologous sequences for 97.25% of Ib53861 and 97.34% of It51184 in the public databases, and the rates of successful validation in a single database ranged from 41.67% to 96.46% (File S2). These results indicate that most of the genes in our datasets are truly transcribed sequences in *I. batatas* and/or *I. trifida*. Furthermore, from the datasets of Ib53861 and It51184, 104,540/94,174 open reading frames (File S1), 25,315/27,090 simple sequence repeats (Files S3–S5), 1,401/1,457 transcription factors (Files S6–S8), 1,656/1,389 long non-coding RNAs (Files S9 and S10), and 5,251/8,901 alternative splicing events (Files S11–S14) were identified. These data provide fundamental information for functional genomics study and molecular breeding in *I. batatas* and comparative biology study between *I. batatas* and *I. trifida*.

### Analysis of long non-coding RNA

Recent studies have shown that lncRNAs act as key regulators in a wide range of biological processes. In the present study, we *in silico* identified 1,656 and 1,389 candidate lncRNAs out of Ib53861 and It51184, respectively (Figs. 2A and 2B; Files S9 and S10). Amongst, 421 *I. batatas* and 355 *I. trifida* transcripts could be recognized as sense, intergenic, intronic, or antisense lncRNAs (Fig. 2C). Notably, there were only 344 common candidate lncRNAs (i.e., homologs in sequences; cutoff: identity >200 bp & >90%) between the identified 1,656 *I. batatas* and 1,389 *I. trifida* transcripts, suggesting remarkable divergence in lncRNA biogenesis and thus their regulatory mechanisms between two species (Fig. 2D). These data suggest that different lncRNA members may be involved in different tissue/organ developmental processes in *I. batatas*.

### Analysis of Alternative splicing

**Figure 3:**
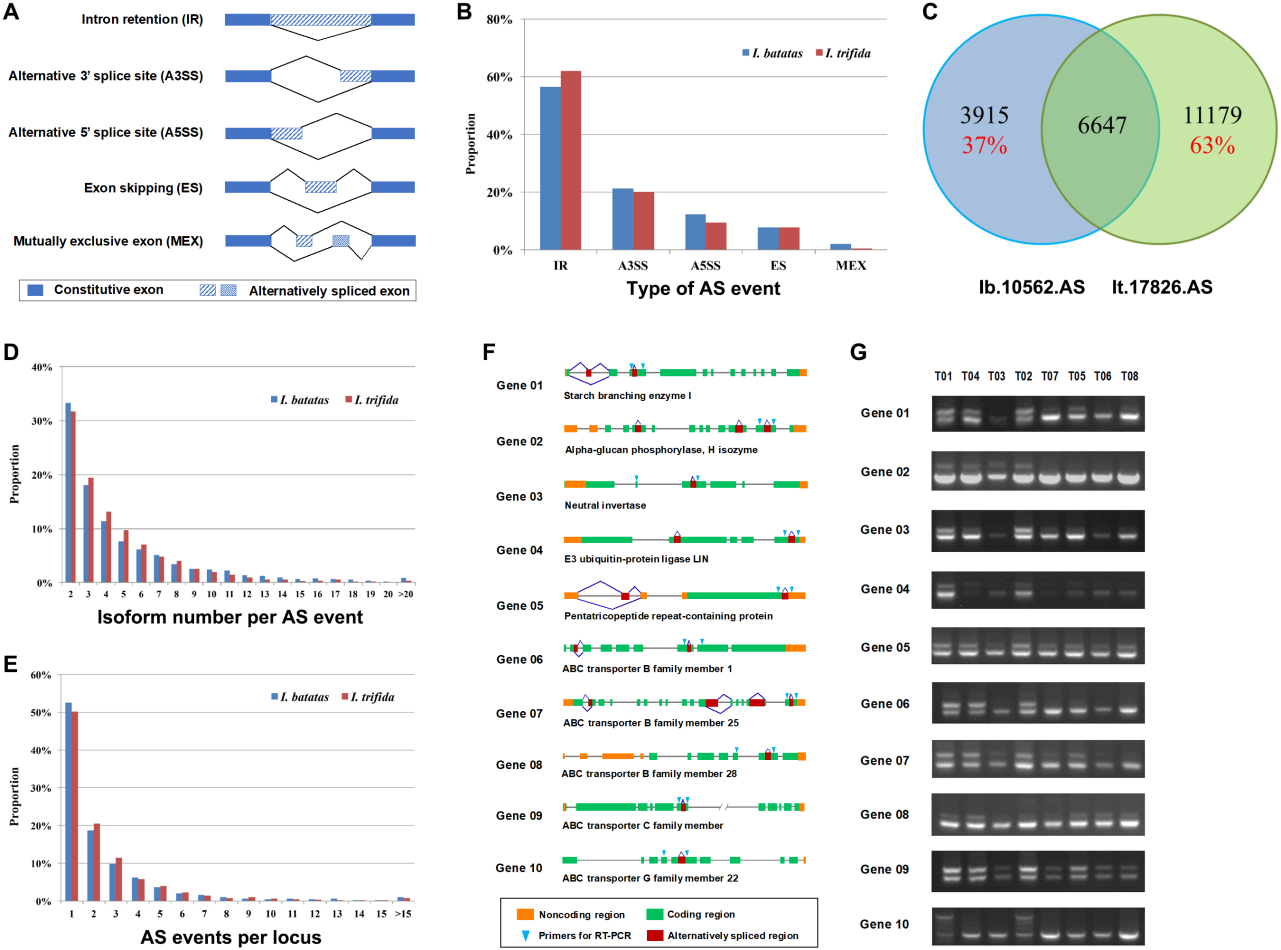
Analysis and validation of alternative splicing (AS). (A) Diagrams showing five major AS types. (B) Proportions of major AS types predicted out of the dataset Ib53861 and It51184. (C) Homolog relationship of isoforms carrying putative AS events between *I. batatas* and *I. trifida*. (D) Proportion distribution of isoform number per AS event in *I. batatas* and *I. trifida*. (E) Proportion distribution of AS events per locus in *I. batatas* and *I. trifida*. (F) Diagram and (G) RT-PCR validation of AS events in ten *I. batatas* genes.

Alternative splicing (AS) is a posttranscriptional regulatory mechanism to increase transcriptome diversity, yet little is known about its roles in the development of tuberous root and the evolution of *I. batatas*. In the present study, we identified 5,251 and 8,901 AS events out of 10,562 and 17,826 transcript isoforms in *I. batatas* and *I. trifida*, respectively (Table 2; Files S11–S14). The AS events were divided into five major types: intron retention (IR), alternative 3′ splice site (A3SS), alternative 5′ splice site (A5SS), exon skipping (ES), and mutually exclusive exon (MEX; Fig. 3A). The proportion of each AS type was comparable between *I. batatas* and *I. trifida* and the majority of AS events were IR in either species (Fig. 3B). Overall, the alternatively spliced isoforms accounted for 32.34% or 38.54% of all isoforms successfully mapped to *I. batatas* scaffolds or *I. trifida* genome (Table 2), which should have largely increased the complexity of transcriptomes in either species. Notably, 37% of the alternatively spliced isoforms in *I. batatas* were not alternatively spliced or not detected in *I. trifida* and so were 63% of the alternatively spliced isoforms in *I. trifida*, suggesting substantial divergence in AS biogenesis and thus their regulatory mechanisms between two species (Fig. 3C). The isoform number per AS event ranged from 2 to 35 (mean, 4.98) in *I. batatas* and from 2 to 46 (mean, 4.55) in *I. trifida* (Table 2; Fig. 3D). In total, 2,074 loci in *I. batatas* and 3,640 in *I. trifida* were involved in the detected AS events (Table 2). The maximal number of AS events per locus was 45 (mean, 2.57) in *I. batatas* and 38 (mean, 2.45) in *I. trifida* (Table 2; Fig. 3E).

**Table 2:** Summary of alternative splicing analysis.

	*I. batatas*	*I. trfida*
Number of isoforms of the datasets	53,861	51,184
Number of isoforms mapped to genome sequences	32,660	46,249
Number of isoforms (with multiple involvements) in AS events	26,146	40,473
Number of isoforms (with one involvement) in AS events	10,562	17,826
Number of detected AS events	5,251	8,901
Maximal number of isoforms in a single AS event	35	46
Mean number of isoforms per AS event	4.98	4.55
Number of loci occuring AS events	2,047	3,640
Maximal number of AS events in a single locus	45	38
Mean number of AS events per locus	2.57	2.45
Mean number of isoforms (with one involvement) per locus	5.16	4.90
Proportion of isofroms undergone AS	32.34%	38.54%
Number of estimated loci in the datasets	10,439	10,452

To assess our large-scale predictions of AS events, we manually examined 40 genes that were predicted as containing AS events and found 8 of them were likely false candidates. We then designed primers to examine 16 AS events, each of which located in one gene, by RT-PCR across eight tissues of an *I. batatas* variety (*Xushu22*), and successfully confirmed 10 of them (Figs. 3F and 3G). According these results, we concluded that at least 50% of our AS predictions were valid. Given that we only examined one of multiple AS events in each gene and only in one *I. batatas* variety, our data should be underestimated. Therefore, our large-scale AS analysis has provided a useful resource for studying biological functions of transcript isoforms and the regulatory mechanism of alternative splicing during the evolution of *I. batatas*.

For example, starch-branching enzymes (EC 2.4.1.18) are one of key enzymes involved in plant starch biosynthesis and sugar metabolism  (Zeeman, Kossmann & Smith, 2010). In our analysis, we detected multiple AS events (i.e., one ES and one IR events) in a putative *I. batatas* starch-branching enzyme I and verified two AS isoforms, whose expression changed over different tissues (Fig. 3G, Gene01). In aboveground tissues (i.e., T01 to T04) and fibrous roots (i.e., T05), the two isoforms were expressed at a similar level; whereas in tuberous roots (i.e., T06 to T08), the smaller isoform were specifically expressed (Fig. 3G, Gene01). Plant alpha-glucan phosphorylases, also named as starch phosphorylase (EC 2.4.1.1), are another important family of enzymes involved in carbohydrate metabolism (Rathore et al., 2009). Our results revealed distinct splicing mechanisms existed between aboveground and belowground tissues in the examined *I. batatas* alpha-glucan phosphorylase (Fig. 3G, Gene02). In addition, divergent gene-expression and splicing patterns were also observed in other investigated genes including a neutral invertase, an E3 ubiquitin-protein ligase, a pentatricopeptide repeat-containing protein, and a few ABC transporters (Fig. 3G, Gene03–10). These data revealed that alternative splicing and thus transcriptome regulation might play important roles during the development of tuberous roots in *I. batatas*.

## Discussion

Understanding the genetic basis and evolutionary scenario underlying agronomically important traits is one of central research themes in the hexaploid crop *I. batatas*. However, achieving this goal is doomed to be challenging because of the complexity of its genome structure (Isobe, Shirasawa & Hirakawa, 2017). In the present study, we applied a hybrid sequencing approach to generate and analyze large-scale full-length or long-read transcripts and their expression profiles in *I. batatas*. Our study would be beneficial to the *I. batatas* research community at least in the following aspects: gene cloning, gene family analysis, development of cDNA-derived marker for breeding, gene model prediction, genome assembly, and study of genetic variation within or among species. For example, we have demonstrated an example of fast gene cloning and gene family analysis basing on our transcriptome datasets  (Ding et al., 2017). Overall, our study has provided a fundamental resource for functional genomics study in *I. batatas*, which would certainly facilitate genetic dissections of the origin of tuberous root as well as other traits.

AS commonly occurs in eukaryotes. In humans, more than 90% of genes were found to be alternatively spliced and the predominant AS type was exon-skipping (Wang et al., 2008). In higher plants, the AS frequency in intron-containing genes approximately ranged from 33% to 60% with intron retention as the major type (Filichkin et al., 2010; Zhang et al., 2010; Shen et al., 2014; Thatcher & Li, 2014). In our study, we observed an overall AS frequency of 32.34% in *I. batatas* isoforms (Table 2). Considering about 30.03% of isoforms contained a single exon in our dataset, the AS frequency in intron-containing isoforms in *I. batatas* was approximately 46.22%. The estimated AS frequency in intron-containing isoforms in *I. trifida* was 51.18%, a little bit higher than that of *I. batatas*. The major AS type was intron retention in either *I. batatas* or *I. trifida*, similar as observed in other plants. These data highlighted the prevalence of AS in both *I. batatas* and *I. trifida*, which would certainly increase the complexity of their transcriptomes. In addition, we also examined the AS pattern across eight tissues in 10 *I. batatas* genes and found that many isoforms exhibited a tissue-specific expression pattern (Fig. 3G). These results imply that the generation of AS isoforms in a tissue-dependent manner have contributed substantially to organ/tissue development and species evolution in *I. batatas*.

AS and gene/genome duplication are two fundamental biological processes contributing to transcriptome and proteome diversity. The relationship between these two evolutionary mechanisms remains debatable. Some studies have reported that the AS frequency decreased after gene duplication and genome polyploidization (Kopelman, Lancet & Yanai, 2005; Su et al., 2006). In contrast, some other reports argued that the evolutionary relationship between AS and gene/genome duplication was more complex and must be cautiously anticipated (Lin et al., 2008; Roux & Robinsonrechavi, 2011; Iñiguez & Hernández, 2017). In this study, our transcriptome-wide AS analysis revealed comparable AS patterns between *I. batatas* and *I. trifida*, in terms of mean number of isoforms per AS event or per locus, mean number of AS events per locus, and proportion of isoforms undergone AS (Table 2; Fig. 3). These data showed that the overall AS frequency (not between specific duplicated gene pairs) was not evidently decreased after the genome hexaploidization in *I. batatas*.

## Conclusions

Although *I. batatas* is a global crop of great agronomic importance, advances in its functional genomics study and molecular breeding remain limited because of the complexity of its genome. Here we report the first collections and analyses of large-scale full-length or long-read transcripts in *I. batatas* and its putative diploid ancestor *I. trifida* using single-molecule real-time sequencing. By performing comprehensive intraspecific and interspecific sequence analyses, we provide a valuable resource for genetic marker development, gene discovery, and gene function study in *I. batatas*, as well as comparative biology study between *I. batatas* and *I. trifida*. Furthermore, we analyzed transcriptome-wide long non-coding RNA and alternative splicing, which revealed tissue-specific-expressed transcript isoforms and the importance of transcriptome regulation during the speciation and domestication of *I. batatas*.

##  Supplemental Information

10.7717/peerj.7933/supp-1 File S1 Number and length distributions of predicted open reading frames

10.7717/peerj.7933/supp-2 File S2 Functional assignment

10.7717/peerj.7933/supp-3 File S3 Summary of predictions of simple sequence repeats (SSRs)

10.7717/peerj.7933/supp-4 File S4 Information of SSRs predicted from Ib53861

10.7717/peerj.7933/supp-5 File S5 Information of SSRs predicted from It51184

10.7717/peerj.7933/supp-6 File S6 Identification of transcription factors

10.7717/peerj.7933/supp-7 File S7 List of transcription factors identified from Ib53861

10.7717/peerj.7933/supp-8 File S8 List of transcription factors identified from It51184

10.7717/peerj.7933/supp-9 File S9 List of lncRNAs predicted from Ib53861

10.7717/peerj.7933/supp-10 File S10 List of lncRNAs predicted from It51184

10.7717/peerj.7933/supp-11 File S11 Information of AS events in Ib53861

10.7717/peerj.7933/supp-12 File S12 Information of AS events in Ib53861 with GFF format

10.7717/peerj.7933/supp-13 File S13 Information of AS events in It51184

10.7717/peerj.7933/supp-14 File S14 Information of AS events in It51184 with GFF format

10.7717/peerj.7933/supp-15 Supplemental Information 15 Long-read transcript sequences of Ipomoea batatas, part one

10.7717/peerj.7933/supp-16 Supplemental Information 16 Long-read transcript sequences of Ipomoea batatas, part two

10.7717/peerj.7933/supp-17 Supplemental Information 17 Long-read transcript sequences of Ipomoea trifida, part one

10.7717/peerj.7933/supp-18 Supplemental Information 18 Long-read transcript sequences of Ipomoea trifida, part two

## Additional Information and Declarations

### DNA Deposition

The following information was supplied regarding the deposition of DNA sequences: The assembled sequences of full-length transcriptomes are available at DDBJ/EMBL/GenBank:GHYO00000000.

### Data Availability

The following information was supplied regarding data availability: The PacBio SMRT reads and the Illumina short reads are available at the Genome Sequence Archive of Beijing Institute of Genomics, Chinese Academy of Sciences (https://bigd.big.ac.cn/gsa/browse/CRA000288).
