# Supplementary material for: Single-molecule real-time sequencing identifies massive full-length cDNAs and alternative-splicing events that facilitate comparative and functional genomics study in the hexaploid crop sweet potato
Source: PeerJ. 2019 Nov 15;7:e7933. doi: 10.7717/peerj.7933 (PMC6859871; doi:10.7717/peerj.7933)
Supplement: File S1 [file peerj-07-7933-s001.pdf]

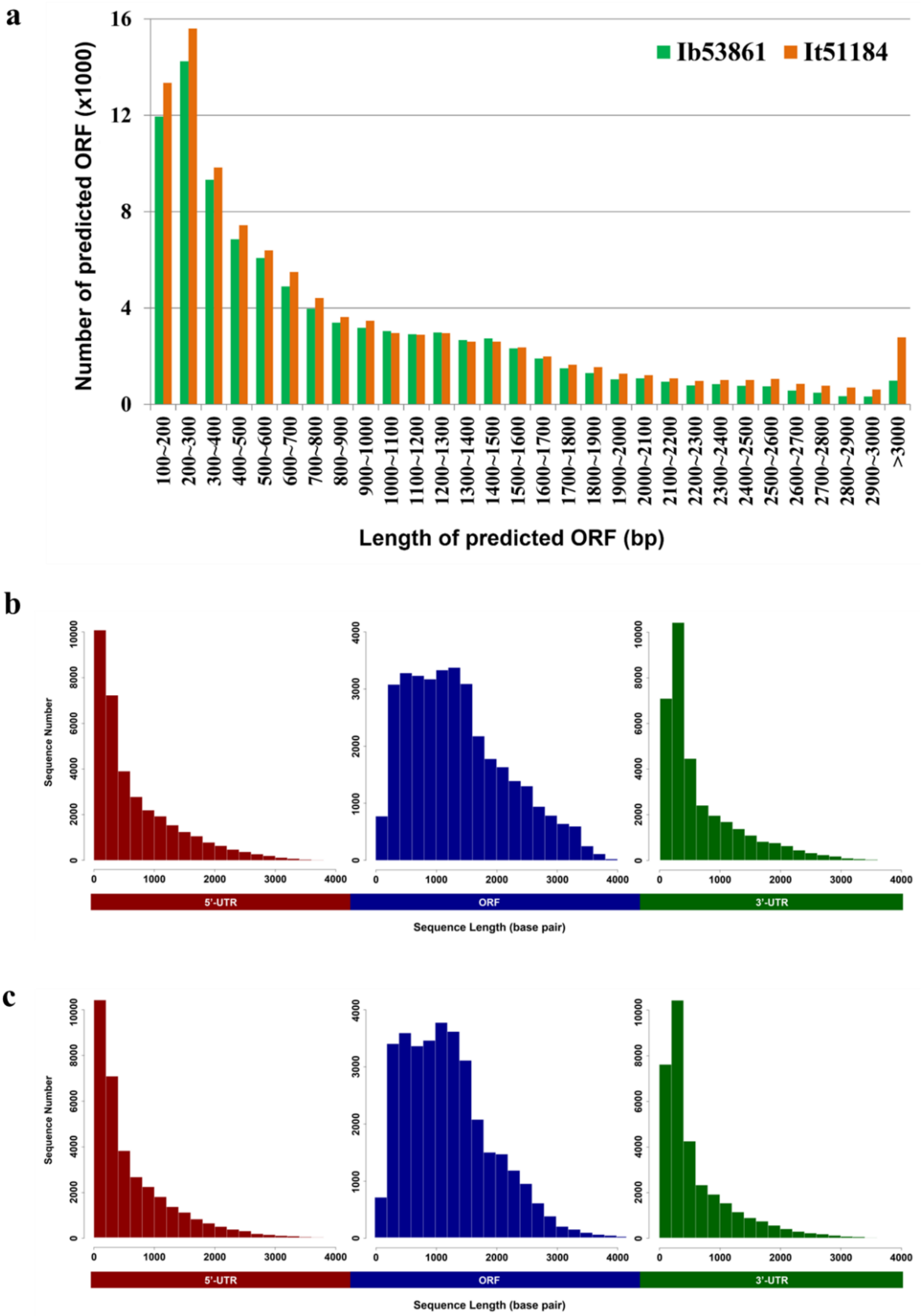

Additional file 1. Number and length distributions of predicted open reading frames. (**a**) Number and length distributions of predicted open reading frames (ORFs) in 53,861 non-redundant transcripts in sweet potato (Ib53861) and 51,184 in *I. trifida* (It51184). (**b,c**) Number and length distributions of characteristic components of 34,963 non-redundant full-length transcripts in sweet potato (**b**) and 33,637 in *I. trifida* (**c**), UTR, untranslated region; ORF, open reading frame.
