# Supplementary material for: Single-molecule real-time sequencing identifies massive full-length cDNAs and alternative-splicing events that facilitate comparative and functional genomics study in the hexaploid crop sweet potato
Source: PeerJ. 2019 Nov 15;7:e7933. doi: 10.7717/peerj.7933 (PMC6859871; doi:10.7717/peerj.7933)
Supplement: File S2 [file peerj-07-7933-s002.pdf]

Additional file 2. Functional assignment

Annotated databases	Ib53861		It51184	
	Transcript Number	Percentage	Transcript Number	Percentage
**COG**	22,571	41.91%	21,794	42.58%
**GO**	29,429	54.64%	29,557	57.75%
**KEGG**	22,442	41.67%	21,589	42.18%
**KOG**	33,480	62.16%	32,621	63.73%
**Pfam**	44,406	82.45%	42,178	82.40%
**Swiss-Prot**	40,094	74.44%	38,190	74.61%
**TrEMBL**	51,611	95.82%	49,185	96.09%
**NR**	50,346	93.47%	49,371	96.46%
**All annotated**	52,378	97.25%	49,825	97.34%
**All analyzed**	53,861	100.00%	51,184	100.00%
