# Supplementary material for: Single-molecule real-time sequencing identifies massive full-length cDNAs and alternative-splicing events that facilitate comparative and functional genomics study in the hexaploid crop sweet potato
Source: PeerJ. 2019 Nov 15;7:e7933. doi: 10.7717/peerj.7933 (PMC6859871; doi:10.7717/peerj.7933)
Supplement: File S3 [file peerj-07-7933-s003.pdf]

Additional file 3. Summary of prediction of simple sequence repeats (SSRs)

	**Ib53861**	**It51184**
Total number of sequences examined	53,841	51,164
Total size of examined sequences (bp)	130,402,110	112,068,291
Total number of identified SSRs	25,319	27,090
Number of SSR containing sequences	17,340	17,127
Number of sequences containing more than 1 SSR	5,401	5,448
Number of SSRs present in compound formation	2,542	4,700
Mono-nucleotide	11,640	14,031
Di-nucleotide	6,942	6,571
Tri-nucleotide	6,092	5,784
Tetra-nucleotide	427	432
Penta-nucleotide	124	154
Hexa-nucleotide	94	118
