# Supplementary material for: Single-molecule real-time sequencing identifies massive full-length cDNAs and alternative-splicing events that facilitate comparative and functional genomics study in the hexaploid crop sweet potato
Source: PeerJ. 2019 Nov 15;7:e7933. doi: 10.7717/peerj.7933 (PMC6859871; doi:10.7717/peerj.7933)
Supplement: File S6 [file peerj-07-7933-s006.pdf]

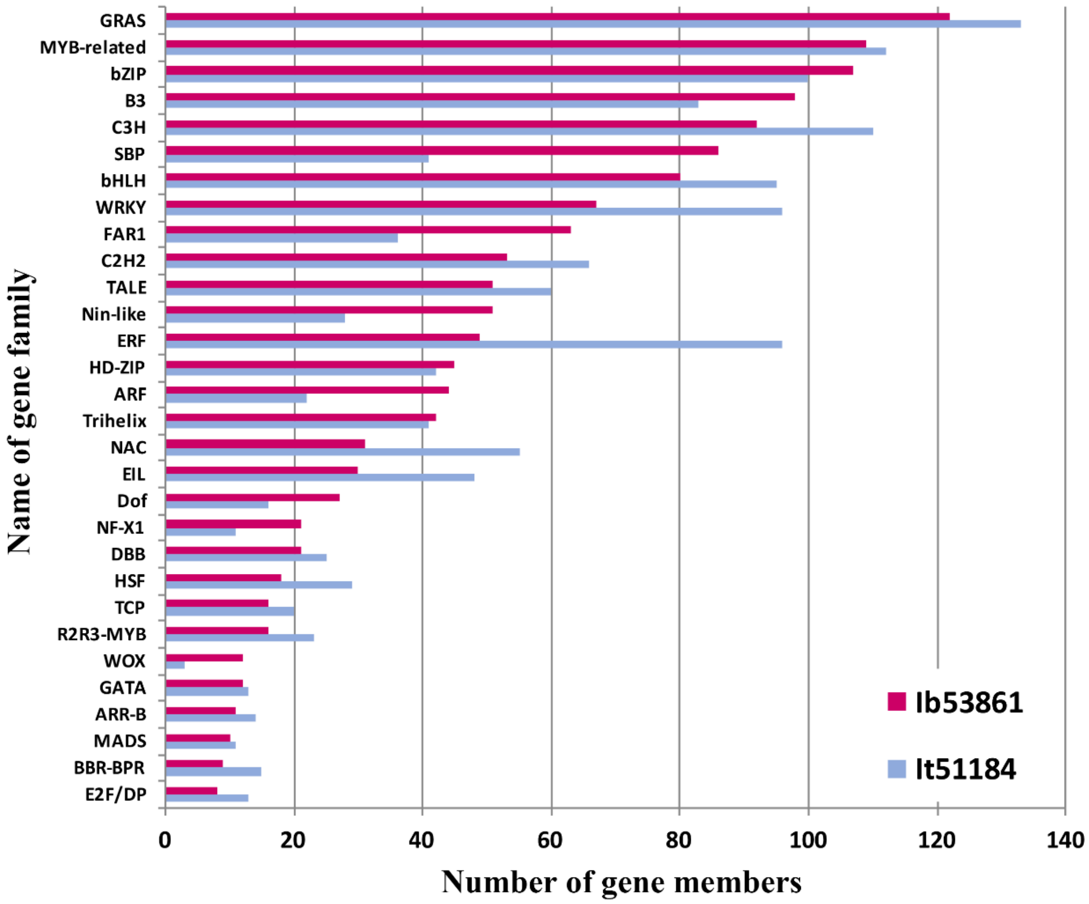

Additional file 6. Identification of transcription factors from our full-length transcriptomes. Family names and numbers of putative transcription factors identified from the datasets Ib53861 and It51184.
